# Long-term follow-up of full-thickness skin grafting in giant incisional hernia repair: a randomised controlled trial

**DOI:** 10.1007/s10029-021-02544-z

**Published:** 2021-12-14

**Authors:** V. Holmdahl, B. Stark, L. Clay, U. Gunnarsson, K. Strigård

**Affiliations:** 1 Department of Surgical and Perioperative Sciences, Surgery, Sunderby Research Unit, Umeå University, Sjukhusvägen 10, 95442 Södra Sunderbyn, Sweden; 2 Department of Plastic and Reconstructive Surgery, Karolinska University Hospital, MK1 Karolinska Institute, Stockholm, Sweden; 3 Department of Clinical Science and Education Södersjukhuset, Karolinska Institute, Stockholm, Sweden; 4 Department of Surgery, Södersjukhuset, Stockholm, Sweden; 5 Department of Surgical and Perioperative Sciences, Surgery, Umeå University, Umeå, Sweden

**Keywords:** Giant incisional hernia, Full-thickness skin graft, Randomised controlled trial, Quality-of-life

## Abstract

**Purpose:**

Conventional repair of a giant incisional hernia often requires implantation of a synthetic mesh (SM). However, this surgical procedure can lead to discomfort, pain, and potentially serious complications. Full-thickness skin grafting (FTSG) could offer an alternative to SM, less prone to complications related to implantation of a foreign body in the abdominal wall. The aim of this study was to compare the use of FTSG to conventional SM in the repair of giant incisional hernia.

**Methods:**

Patients with a giant incisional hernia (> 10 cm width) were randomised to repair with either FTSG or SM. 3-month and 1-year follow-ups have already been reported. A clinical follow-up was performed 3 years after repair, assessing potential complications and recurrence. SF-36, EQ-5D and VHPQ questionnaires were answered at 3 years and an average of 9 years (long-term follow-up) after surgery to assess the impact of the intervention on quality-of-life (QoL).

**Results:**

Fifty-two patients were included. Five recurrences in the FTSG group and three in the SM group were noted at the clinical follow-up 3 years after surgery, but the difference was not significant (*p* = 0.313). No new procedure-related complication had occurred since the one-year follow-up. There were no relevant differences in QoL between the groups. However, there were significant improvemnts in both physical, emotional, and mental domains of the SF-36 questionnaire in both groups.

**Conclusion:**

The results of this long-term follow-up together with the results from previous follow-ups indicate that autologous FTSG as reinforcement in giant incisional hernia repair is an alternative to conventional repair with SM.

**Trial Registration:**

The study was registered August 10, 2011 at ClinicalTrials.gov (ID NCT01413412), retrospectively registered.

## Introduction

Incisional hernia (IH) is a common complication affecting around 10% of patients after major abdominal surgery [1]. In many cases, IH has a negative effect on quality-of-life (QoL) and restricts performance of daily activities due to pain and discomfort [2]. Potentially life-threatening strangulation where blood supply to the hernial content is compromised, is a feared complication of IH. Though many patients with IH are asymptomatic, some require surgical repair. Complexity of the surgical procedure is primarily related to the size of the hernia aperture, and a large IH can require special surgical techniques for reconstruction. Giant IH is often defined as a hernia with a transverse aperture size wider than 10 cm (size grade three according to the EHS classification of IH) [3].

Modern repair of IH (diameter > 1 cm) involves reinforcement with a prosthetic mesh. This has reduced the number of recurrences seen when performing suture repair [4]. However, in the subgroup of patients with a giant IH, hernia recurrence occurs in up to 33% of patients even after mesh reinforcement [5, 6]. Furthermore, synthetic mesh procedures are associated with more complications than non-mesh methods [7], and there is a correlation between hernia aperture size and both frequency and severity of complications [8].

An increasing concern after hernia surgery is the occurrence of long-term complications, and these must be considered when evaluating results after hernia repair [7, 9]. An example is chronic pain after implantation of non-absorbable mesh [10].

Autologous full-thickness skin graft (FTSG) may be an alternative reinforcement material offering better tissue integration where synthetic mesh causes some degree of foreign body reaction and scarring. Potentially, this advantage over synthetic mesh could offer a more comfortable alternative, less prone to pain, recurrence, and complications. Autologous full-thickness skin grafting was first tried in the early twentieth century [11, 12], but after the introduction of modern synthetic mesh, FTSG fell into obscurity and has not been compared with materials that are now gold standard reinforcement in other types of hernia [13–15]. In view of the increasing awareness of long-term complications as an important outcome, and the poor results of current methods of repair for giant IH, a review of the FTSG technique is warranted.

This study is a long-term follow-up of a randomised controlled trial comparing the use of full-thickness skin grafting to synthetic mesh as reinforcement in the repair of giant IH.

Short-term complications at a 3-month follow-up were the main outcome measures of the trial published in 2017 [16]. Furthermore, a one-year follow-up focusing on abdominal muscle strength was also published [17]. The long-term follow-up in this study focuses on health-related QoL and pain, as well as a clinical examination evaluating any recurrence.

## Materials and methods

### Study design

A randomised controlled trial was conducted at two university hospitals specialising in abdominal wall surgery. Inclusion and exclusion criteria are presented in Table 1. Patients included in the study were randomised to repair with FTSG in the onlay position or synthetic mesh in the sublay position where possible, otherwise in the onlay position.

**Table 1:** Inclusion and exclusion criteria

Inclusion	Exclusion
Incisional hernia > 10 cm wide	Ongoing immunosuppressive treatment
Above 18 years of age	Ongoing pregnancy or breastfeeding
	Smoking < 3 months prior to surgery

In the FTSG group, the transplant was harvested adjacent to the midline incision, including scar tissue and skin overlying the hernia. The FTSG was then prepared with removal of all subcutaneous tissue and knife-meshed with multiple incisions, 5–10 mm of length. The anterior rectus fascia was exposed, and the hernia defect was closed with a continuous polydioxanone monofilament suture, size 0. The FTSG was then placed on the fascia and anchored with single, interrupted, absorbable monofilament sutures, size 4–0.

In the synthetic mesh group, the goal was to place a lightweight polypropylene mesh in the sublay position. Initially the hernia sac was exposed through a midline incision followed by exposure of the retro-rectus space to enable placement of a mesh with an overlap of at least 5 cm. The hernia defect was closed with a continuous polydioxanone monofilament suture size 2–0, and the mesh was placed without any anchoring sutures. The anterior rectus fascia was then closed with a continuous polypropylene, monofilament suture, size 0. If the retro-rectus space was judged too obliterated or otherwise too risky to access, a heavyweight polypropylene mesh was placed in the onlay position after fascial closure with continuous polypropylene, monofilament suture, size 0. In these cases, the mesh was anchored with double rows of polydioxanone monofilament sutures, size 2–0.

These procedures have been described in greater detail in previous publications [16, 17]. The patients, nursing staff, and surgeons performing the follow-ups were blinded to the procedure performed. All procedures were conducted by at least one of two consultant surgeons with many years of experience in hernia surgery.

Based on a predicted 3-month complication rate of 50% in the synthetic mesh group and 20% in the FTSG group, 50 patients were needed to obtain 80% power and 95% significance. The sample size was thus not calculated on the outcomes of this trial.

### Clinical follow-up

A clinical examination was performed approximately 3 years after the repair where the patient’s general clinical status, any recurrence, and any surgical complication during the postoperative period were assessed. The aesthetical results on the abdominal region were documented and the patients were screened for prevalence of pain and how satisfied they were with the surgical intervention. If there was uncertainty regarding the presence of a recurrence, computerised tomography was performed to confirm the diagnosis. Bulging and pseudo-hernias were not considered as a recurrence.

### Questionnaires

To evaluate health-related QoL and pain, all patients were asked to answer three questionnaires before surgery as well as at the three-year follow-up. The same questionnaires were then sent to all surviving patients September 2020. Patients who did not answer the first time were sent two reminders a few weeks apart. The following questionnaires were used:

- SF-36, developed by Medical Outcome Trust, is a widespread and frequently used quality-of-life questionnaire comprising 36 questions covering eight health concepts: physical functioning, role limitations a. due to physical health problems and b. emotional problems, vitality, bodily pain, social functioning, mental health, and general health. The SF-36 is validated [18] and available in Swedish [19].
- VHPQ is a validated questionnaire specially designed to evaluate pain following ventral hernia repair and developed by our research group. It focuses on pain related to behaviour [20].
- EQ-5D, developed by the EuroQol group is a generic, accessible, and compact measure of health status. The respondent classifies his or her health in five dimensions (mobility, self-care, usual activities, pain/discomfort, and anxiety/depression) along with a visual analogue scale (VAS) estimate of overall state of health.

### Statistics

All data were gathered in Access™-databases (Microsoft, Redmond, Washington, USA). Statistical analyses were carried out on SPSS v.27 IBM Corp., Armonk, NY, USA). Continuous variables were tested with Student’s t test and dichotomous variables were tested using Chi-square statistics or Fischer’s exact test when Chi-square criteria were not met, and with McNemar’s test for paired data.

### Ethics

This study was approved by the Regional Board of Ethics at the Karolinska Institute, Stockholm (reference numbers: 2009/227-31/3 and 2012/1775-32).

## Results

**Fig. 1:**
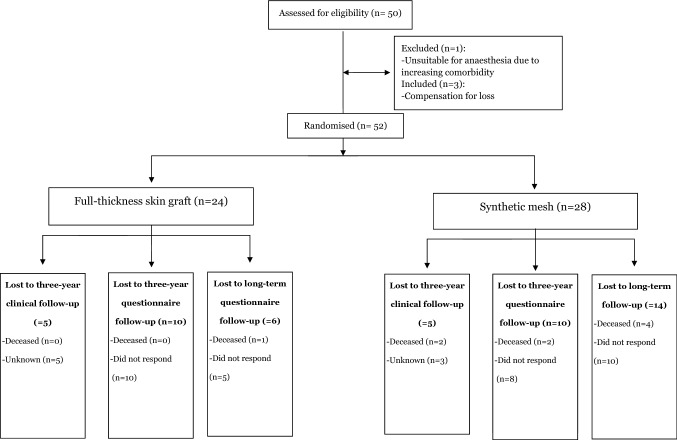
Consort flow diagram

Fifty-two patients were included in the study, and the numbers of patients attending the follow-ups are presented in the flow chart in Fig. 1. Cause of death in patients who died during the follow-up period showed no correlation with the study intervention. There were 27 males and 25 females with an average age of 64 years at the time of surgery. Average BMI was 31 and hernia width 13.9 cm, with no significant differences according to treatment allocation.

The mean follow-up for clinical assessment was 3.1 years (range 2.32–3.61) with 42 patients examined, corresponding to 84% of those still alive at the time of follow-up. Since the 1-year follow-up, three more recurrences had occurred in the FTSG group and one in the synthetic mesh group, the difference, however, remained insignificant (*p* = 0.313). The recurrences were in general small and caused few symptoms, it was not noted whether a recurrence was central or peripheral. There were no reports of new surgical complications developing after the one-year follow-up. No cyst formation in the FTSG group was noted, neither clinically nor with computerized tomography.

The mean follow-up times for the QoL questionnaires were 3.4 years (range 3.1–4 years) for the 3-year follow-up and 9.3 years (range 7.3–10.5 years) for the long-term follow-up. Response rates for the 3-year follow-up and the long-term follow-up were 64% and 70%, respectively, accounting for deceased participants. A summary of the main findings from the VHPQ questionnaire is presented in Table 2. Two entities having considerable clinical relevance in the VHPQ questionnaires were whether the respondents experienced pain “right now” or “during the last week”. The degree of symptoms was rated on a 7-grade scale where grades 1 and 2 were considered clinically irrelevant. To simplify interpretation, results were dichotomized according to this breakpoint. Notably, the number of patients complaining from significant pain both “right now” and “last week” decreased in both groups at the 3-year follow-up, but more so in the FTSG group. This decrease remained at the long-term follow-up, but there was neither any significant differences between the groups nor any difference within the groups regarding any of the VHPQ questions.

**Table 2:** VHPQ Questionnaire

	FTSG						Synthetic mesh					
	Preop	Missing	3 years	Missing	Long-term	Missing	Preop	Missing	3 years	Missing	Long-term	Missing
Pain right now ⩽ 2	6 (30%)	0	10 (71%)	0	13 (72%)	0	14 (61%)	0	13 (72%)	0	10 (71%)	0
Pain right now > 2	14		4		5		9		5		4	
Pain last week ⩽ 2	4 (22%)	2	10 (71%)	0	13 (72%)	0	13 (59%)	1	11 (61%)	0	9 (75%)	2
Pain last week > 2	14		4		5		9		7		3	
Functional												
Difficulty rising from chair	7 (50%)	6	2 (40%)	9	1 (25%)	14	7 (58%)	11	4 (44%)	9	2 (33%)	8
Difficulty sitting	6 (43%)	6	3 (60%)	9	1 (33%)	15	6 (50%)	11	3 (33%)	9	1 (17%)	8
Satisfaction												
Satisfied with operation			11 (85%)	1	10 (59%)	1			12 (67%)	0	7 (64%)	3
Would do it again			10 (77%)	1	14 (82%)	1			15 (83%)	0	7 (64%)	3

Yes (Percentage of all answers). Missing = Missing of the total of respondents for each follow-up

In the EQ-5D questionnaire (Table 3), there were improvements in both groups, especially regarding pain and everyday functioning, but no significant differences were observed. The change in self-rated overall health did not change significantly.

**Table 3:** EQ-5D questionnaire

	FTSG			Synthetic mesh		
	Preop	3 years	Long-term	Preop	3 years	Long-term
Mobility	9 (45%)	5 (39%)	8 (44%)	11 (46%)	7 (44%)	5 (36%)
Hygiene	2 (10%)	0	0	1 (4%)	0	3 (21%)
Usual activities	**12 (60%)**	2 (17%)	7 (39%)	**6 (24%)**	4 (27%)	5 (36%)
Pain/discomfort	15 (75%)	6 (55%)	10 (56%)	17 (68%)	10 (63%)	6 (43%)
Anxiety/depression	8 (40%)	2 (17%)	3 (17%)	10 (40%)	7 (41%)	5 (36%)
Self-rated health	64 (17)*	64 (26)*	70.6 (21)*	61.7 (21)*	67 (18)*	69 (23)*

Number of patients rating any grade of problem, percentage of respondents in parentheses. Bold indicates a statistically significant difference in the preoperative assessment between the groups (*p* = 0.014)

*Mean (± Standard deviation)

Condensed data from the SF-36 questionnaire showed a tendency towards sustained general improvement. Some of the physical parameters in the FTSG group and some emotional and mental parameters in the synthetic group improved significantly (Table 4). Apart from the pain dimension at the long-term follow-up, there were no significant differences in outcomes between the two groups.

**Table 4:** SF-36 Questionnaire

	FTSG					Synthetic				
	Preop	3 years	*p* value	Long-term	*p* value	Preop	3 years	*p* value	Long-term	*p* value
Physical functioning	52 (30)	64 (24)	*0,002*	62 (26)	*0,005*	66 (25)	71 (28)	0,425	58 (35)	0,350
Physical role functioning	43 (45)	48 (46)	*0,032*	51 (45)	0,341	36 (38)	68 (42)	*0,009*	60 (50)	0,087
Emotional role functioning	59 (45)	60 (49)	0,339	65 (45)	0,872	58 (47)	72 (43)	*0,027*	74 (40)	0,221
Vitality	56 (26)	57 (32)	0,840	60 (24)	0,274	56 (25)	61 (23)	0,348	55 (21)	0,852
Mental health	73 (23)	78 (31)	0,170	78 (20)	0,284	68 (24)	76 (22)	0,261	80 (21)	*0,034*
Social functioning	68 (29)	68 (36)	0,296	79 (22)	0,161	66 (26)	83 (25)	*0,019*	75 (34)	0,591
Bodily pain	60 (24)	69 (30)	0,062	**83 (22)**	*0,01*	69 (26)	69 (33)	0,695	**62 (31)**	0,618
General Health	56 (26)	52 (24)	0,322	61 (26)	0,499	56 (22)	61 (24)	0,253	52 (19)	0,780

Bold indicates a statistically significant difference between the outcomes between the two treatment groups (*p* = 0.032)

*Mean (± Standard deviation)

## Discussion

The results of this randomised controlled trial indicate that QoL improves after surgical correction of giant IH, and that there is little difference in outcome between FTSG and synthetic mesh as reinforcement of the abdominal wall. In particular, there were improvements in the physical and pain dimensions. The FTSG group scored slightly higher in the SF-36 questionnaire, with statistically significant improvements in physical functioning, physical role functioning, and bodily pain. Due to large inter-individual variability, however, many of the results could not be validated statistically, and it is possible that the number of respondents was too small to provide the power required. This was a long-term follow-up in a relatively aged population with significant comorbidity. Several died during the follow-up period which further reduced the population size.

Since patient suffering motivates surgical repair of an IH, it is to be expected that the largest improvements will be in the QoL dimensions pain and physical functioning. However, physical impairment and chronic pain impacts all aspects of the individual’s QoL and this may explain the SF-36 results in the synthetic mesh group showing improvements in social, mental, and emotional aspects of health. Similar trends were also seen in the emotional aspects of the EQ-5D questionnaire, and the lack of any significant difference between the groups indicates that outcomes were similar.

Most synthetic meshes were placed in the sublay position as this position was considered preferable at the beginning of the trial; a belief that was subsequently confirmed in a meta-analysis [21]. FTSGs were exclusively placed in the onlay position. This position was chosen since knowledge of graft behaviour after implantation in any other position was insufficient at that time. However, more recent studies have shown good FTSG survival in an intraperitoneal position using a murine model, which could indicate that also the sublay position would be feasible [22].

Differences in the positioning of FTSGs and synthetic meshes could have affects the results of this study. It is unclear how the results of studies investigating outcome after different synthetic mesh placements can be applied to FTSG. Some studies suggest that the dissection necessary for sublay positioning causes more trauma to nerves and blood vessels leading to chronic pain [10].

There are two previous publications focusing on QoL after surgical repair of giant ventral hernia. An improvement in QoL was reported, but the study designs were retrospective, and validated questionnaires were not used [23, 24]. This prospective randomised study using validated questionnaires considerably increases our knowledge on how QoL is affected by surgical repair of giant IH. Another strength of this study was the use of three complimentary questionnaires, including a specific ventral hernia assessment, thereby covering overall health as well as specific ventral hernia complaints. However, when using questionnaires, there is a fine balance between the number of questions for maximum return and the number of participants choosing not to participate because it takes too long. The questionnaires in this study only took 10 min or so to answer. Even so, it is possible that some of the patients did not complete follow-up due to the number of questions, thus introducing a risk of bias.

In this study, the average age of patients at the time of surgery was 64 years and many had comorbid conditions apart from the reason for having undergone abdominal surgery that gave rise to the IH. Assessment of overall health can thus be obscured by conditions other than the hernia and its subsequent treatment. This is especially true in the long-term assessment of QoL which in this study was performed after an average of 9 years. Long-term QoL assessment can provide important information on a surgical method’s solidity over time, but it must be interpreted with caution.

A weakness of this study was that the number of patients available did not provide sufficient power to clearly accept or reject the hypothesis. This was partly due to QoL not being the main outcome of the trial, and further aggravated by loss to follow-up.

We cannot say why patients did not complete follow-up or did not answer the questionnaires, and there is always a risk for bias when response rates fall. The response rates of the questionnaires did not differ significantly between the groups. Since the main objective of this study was to compare the two treatments for giant IH, a potential bias caused by the loss to follow-up is most likely evenly distributed because of the randomisation.

The long-term follow-up time ranged between 7.3 and 10.5 years, introduces a potential bias in terms of patient experience from the hernia repair. However, we have considered the dominating part of a possible change during this sampling time emanating from progress of comorbidity rather than from the surgical intervention studied.

The overall recurrence rate in this study was 15.4% which is at the lower range limit compared to previous publications even though the follow-up time of 3 years was relatively long [5, 6]. The slightly higher number of recurrences in the FTSG group was not significant but worth noting, especially since they occurred later than one year after the repair. Recurrences have also been shown to occur several years after hernia repair with synthetic mesh [25].

The use of computerised tomography to detect a recurrent IH is debatable, and there is not enough evidence to recommend it in routine care. Clinical assessment remains the main diagnostic entity for IH [26]. Against this background and considering the known hazards of ionizing radiation, the ethical approval for in this trial only permitted the use of computerised tomography if the clinical diagnose was uncertain.

## Conclusion

Results from this long-term follow-up together with the results from prior follow-ups indicate that repair of giant IH using autologous FTSG as reinforcement could be an alternative to conventional repair with synthetic mesh. The present trial cannot determine which giant IH patients that would benefit the most from FTSG as a reinforcement material, and no recommendations can be made for routine care based on the present evidence. Further studies with appropriate power are needed to fully evaluate potential advantages regarding recurrence rates and QoL outcome.
